# Health-Related Quality of Life in Multiple Myeloma Patients Treated with High- or Low-Dose Lenalidomide Maintenance Therapy after Autologous Stem Cell Transplantation—Results from the LenaMain Trial (NCT00891384)

**DOI:** 10.3390/cancers15215157

**Published:** 2023-10-26

**Authors:** Amelie Boquoi, Aristoteles Giagounidis, Hartmut Goldschmidt, Michael Heinsch, Mathias J. Rummel, Nicolaus Kröger, Elias K. Mai, Judith Strapatsas, Rainer Haas, Guido Kobbe

**Affiliations:** 1 Department of Hematology, Oncology and Clinical Immunology, University Hospital Duesseldorf, 40225 Duesseldorf, Germany; judith.strapatsas@med.uni-duesseldorf.de (J.S.); haas@med.uni-duesseldorf.de (R.H.); kobbe@med.uni-duesseldorf.de (G.K.); 2 Klinik für Onkologie, Hämatologie und Palliativmedizin, Marien Hospital Düsseldorf, Rochusstr. 2, 40479 Düsseldorf, Germany; aristoteles.giagounidis@vkkd-kliniken.de; 3 National Center for Tumor Diseases Heidelberg (NCT), 69120 Heidelberg, Germany; hartmut.goldschmidt@med.uni-heidelberg.de; 4 Department of Internal Medicine V, University of Heidelberg, 69120 Heidelberg, Germany; 5 Helios Klinikum Duisburg, 47166 Duisburg, Germany; michael.heinsch@helios-gesundheit.de; 6 Medizinische Klinik IV, University Hospital, 35392 Giessen, Germany; mathias.rummel@innere.med.uni-giessen.de; 7 University Hospital Eppendorf, 20251 Hamburg, Germany; nkroeger@uke.de; 8 Department of Medicine V, Hematology, Oncology and Rheumatology, University of Heidelberg, 69120 Heidelberg, Germany; elias.mai@med.uni-heidelberg.de

**Keywords:** multiple myeloma, maintenance, lenalidomide, dosage, quality of life, domain

## Abstract

**Simple Summary:**

High-dose therapy with melphalan followed by autologous stem cell transplantation and lenalidomide maintenance has long been the standard of care for newly diagnosed patients with multiple myeloma. However, it is unclear how lenalidomide dosage or experienced side effects govern patients’ quality of life during long-term treatment. The LenaMain trial (NCT00891384) investigated lenalidomide maintenance at a high (25 mg) and a low dose (5 mg) and demonstrated that dosage had no impact on patients’ quality of life. Instead, high baseline scores for global health were maintained throughout the trial without difference between treatment arms which supports the feasibility of continuous lenalidomide treatment with a dose tailored to patients’ quality of life.

**Abstract:**

Introduction: The LenaMain trial (NCT00891384) reported increased progression-free survival with 25 mg of lenalidomide maintenance compared to 5 mg. Here, we report the patient-reported outcomes. Materials and Methods: Scores obtained from the EORTC Quality of Life Questionnaire C30 were analyzed for longitudinal changes from baseline within the groups as well as cross-sectional scores. Results: Compliance rates were high, with 95.7% at baseline and 70% during maintenance. At study entry, scores were high for functioning and low for symptoms. During maintenance, the median global health status/quality of life (GHS/QoL) was constant, without significant differences over time (median GHS/QoL: 68 at baseline and 58 for Len high and 68 for Len low at 2 years) and between treatment arms (mean change < 2). Similarly, most functional scale domains were constant. Notably, diarrhea increased consistently for both treatment arms (baseline: −1.905 (range: −5.78–1.97); end of year 2: 16.071 (range: 5.72–26.42); *p* < 0.05). The subgroup analysis showed that neither disease activity, duration of treatment, nor adverse events affected the health-related quality of life (HR-QoL) or utility. Conclusion: High baseline scores were maintained throughout the trial without significant differences between the Len dosages, which supports continuous treatment with a dose tailored to patients’ HR-QoL.

## 1. Introduction

Patients with multiple myeloma (MM) typically experience a high burden of symptoms, which can significantly impair their health-related quality of life (HR-QoL) [1,2,3]. Moreover, therapy-related side effects can negatively impact HR-QoL [4,5,6,7]. Improvements in the efficacy of MM treatment have made HR-QoL an increasingly important endpoint in clinical trials and a relevant factor for patients and physicians in choosing the most appropriate treatment [8].

High-dose therapy with melphalan followed by autologous stem cell transplant (ASCT) has long been the standard of care for newly diagnosed MM in clinically fit adults [9]. ASCT may lead to the short-term worsening of the HR-QoL but the baseline HR-QoL is generally regained as early as 2 months post-transplant, with long-term improvement in the HR-QoL and pain [10,11]. In contrast to tumor debulking, maintenance therapy aims to delay the disease progression after ASCT, when the disease burden is minimal. Continuous lenalidomide maintenance until progression leads to significantly improved progression-free and overall survival [12,13]. However, treatment-related toxicities, like infections, rash, fatigue, diarrhea/constipation, or neuropathy, could negatively affect patients’ HR-QoL, ultimately leading to discontinuation [14,15,16].

The LenaMain trial (NCT00891384) was an investigator-initiated, randomized, open-label, phase-III trial that randomized patients to either receive lenalidomide at 5 mg (Len low) or 25 mg (Len high) [17]. To date, the LenaMain trial is the only clinical trial to show that a higher dosage of lenalidomide maintenance post-transplant benefits patients by extending the time of remission.

Previous real-world data from the Connect^®^ MM registry, a U.S. non-interventional, prospective registry, have highlighted that lenalidomide maintenance after ASCT has no adverse impact on HR-QoL [18]. But while a maintenance dose of 10–15 mg is approved in most areas as the standard of care, a dose-finding study has never been performed. Therefore, it remains unclear whether a higher dose of lenalidomide maintenance affects HR-QoL. 

HR-QoL assessment using the global health status/quality of life (GHS/QoL) scale of the European Organisation for Research and Treatment of Cancer (EORTC) was a predefined secondary endpoint in the LenaMain trial. Here, we present the full HR-QoL results from cross-sectional and longitudinal analyses of the secondary and exploratory HR-QoL endpoints. Furthermore, this is the first time that the HR-QoL is compared between two cohorts receiving different dosages of lenalidomide maintenance (25 and 5 mg). 

## 2. Methods

### 2.1. Study Design and Treatment

The LenaMain trial recruited patients from 4 June 2009 to 1 February 2015 in six hospitals in Germany. Details were previously published [17]. Briefly, patients were randomized 1:1 to either receive lenalidomide at a 5 mg (Len-low) or 25 mg (Len-high) maintenance treatment (Len MT) after first-line therapy with autologous stem cell transplant. Patients in both treatment arms first entered a uniform consolidation period and received 6 cycles of lenalidomide at 25 mg (for 21 days every 28 days), after which they started their respective maintenance therapies.

### 2.2. HR-QoL Assessments and Endpoint

The EORTC Quality of Life Questionnaire, QLQ-C30, is the most frequently used instrument for cancer-specific HR-QoL assessment in myeloma patients [19,20,21] and includes 30 items comprising one global health status (GHS) scale, five functional scales (physical, role, emotional, cognitive, and social functioning), three symptom scales (fatigue, nausea and vomiting, and pain), and six single items (dyspnea, insomnia, appetite loss, constipation, diarrhea, and financial difficulties). Higher scores represent better GHS and functioning but also greater (i.e., worse) symptoms. The primary patient-reported outcome (PRO) hypothesis presumed similar HR-QoL for the two different lenalidomide doses during maintenance. The minimally important difference (MID) threshold for clinically meaningful change from baseline was defined a priori based on the published literature, with a value of ≥5 points considered as clinically significant [22]. Evidence-based guidelines were used to interpret the longitudinal change from baseline within the groups [23] as well as the cross-sectional scores [24] (see Appendix A for further details). Patients completed the questionnaires on day 1 of each cycle. A total of 194 patients were enrolled in six German centers and 188 patients were randomized to receive Len maintenance.

The study was conducted in accordance with the Declaration of Helsinki and approved by the Institutional Review Board of Heinrich Heine University (Approval Date: 16 January 2009, Approval Number: MC-LKP-300).

## 3. Results

### 3.1. Patient Population

From 4 June 2009 to 1 February 2015, 194 patients were enrolled in six German centers and 188 patients were randomized to receive lenalidomide maintenance. The patient demographics were balanced across the treatment groups in terms of baseline characteristics such as age, gender, risk scores, and prior therapy, without statistically significant differences. The median age at enrolment was 59 years. The QLQ-C30 domain values and utility values were balanced between the arms at both the consolidation baseline and maintenance baseline without statistically significant differences, which suggests no significant departure as a result of the differing effects of treatment with lenalidomide at 25 mg during the consolidation period (Table 1). 

### 3.2. Compliance

**Figure A1:**
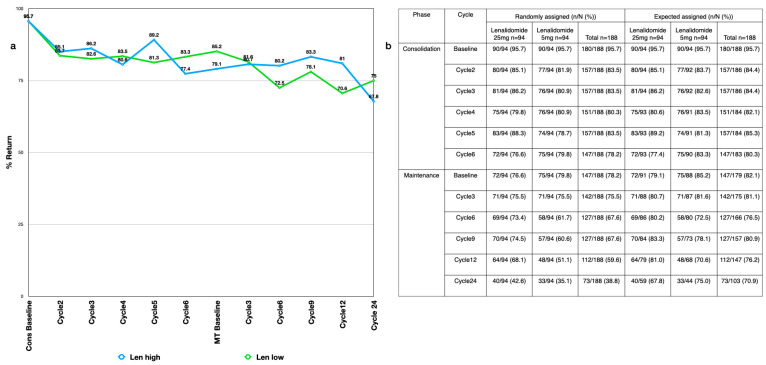
(**a**) Compliance by cycle for the expected assigned population (**b**) return rate with n = actual numbers returned, N = numbers eligible, both in %. A summary of missing observations of the QLQ-C30 domains overall and by treatment arm shows the number of records at baseline and at each specified cycle compared to both the number of randomly assigned patients and the approximate expected number of patients. This helps to demonstrate the amount of data available for the analyses, and gives an idea of return rates.

The compliance rates were high, with 95.7% of all randomly assigned patients completing the QLQ-C30 questionnaire at baseline (Appendix B Figure A1a). During the consolidation period, the compliance rates remained high, with a return of more than 80% of the expected responses. During the maintenance period, the compliance rates remained close to 70% or above. Slight differences between the treatment groups were expected after correction for dropouts due to progressive disease (Appendix B Figure A1b).

### 3.3. Quality of Life during the Consolidation Phase

#### 3.3.1. Global Health Status/Quality of Life (GHS/QoL) and Utility Values

**Figure A2:**
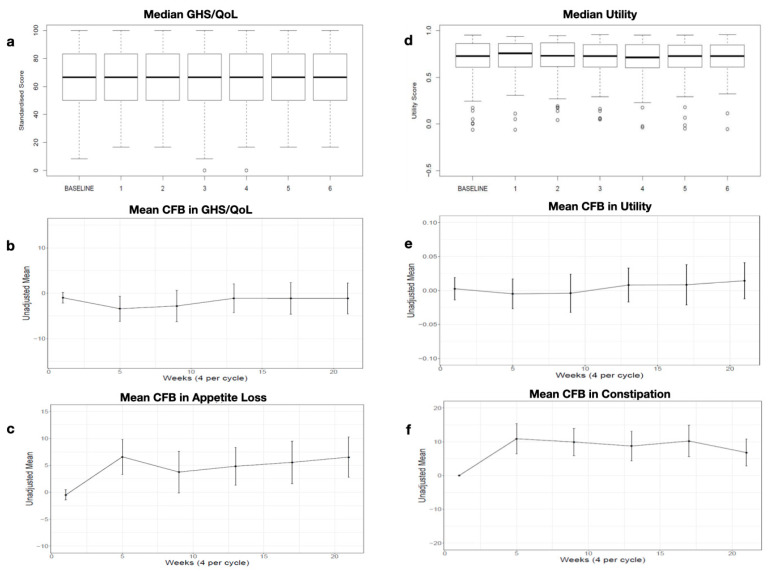
Consolidation (**a**) Median GHS/QoL by cycle (**b**) Mean CFB in GHS/QoL by cycle (**c**) Mean CFB in Appetite Los by cycle (**d**) Median Utility by cycle (**e**) Mean CFB in Utility bay cycle (**f**) Mean CFB in Constipation.

The GHS/QoL was high at the consolidation baseline, with a mean of 65.78 (range: 0–100). The median GHS/QoL scores were constant over 6 months of consolidation (Appendix C Figure A2a). The mean change from baseline (CFB) over time was very small, and there was no obvious trend over the six cycles (baseline: −0.98 (range: −2.16–0.2), *p* > 0.05; cycle 6: 1.123 (range: −4.54–2.3), *p* > 0.05; Appendix C Figure A2b).

Similarly, the utility was high at the consolidation baseline, with a mean of 0.7 (range: 0–1). Little variation was seen in the median utility values across the consolidation cycles (Appendix C Figure A2d). The mean CFB over time was very small, and there was no obvious trend over the six cycles (baseline: 0.003 (range: 0.01–0.02), *p* > 0.05; cycle 6: 0.014 (range: −0.01, 0.04), *p* > 0.05; Appendix C Figure A2e).

#### 3.3.2. EORTC QLQ-C30 Domain Scores

At study entry, the EORTC QLQ-C30 scores were high for functioning and low for symptoms. Most CFBs in the domains were less than 5 points and therefore not considered relevant. However, some domains show a few noteworthy changes over time: Appetite loss symptom scores appear to generally become worse, with an average increase of approximately 7 points at cycle 6, surpassing the MID (baseline: −0.49 (range: −1.47–0.49, *p* > 0.05); cycle 6: 6.527 (range: 2.76–10.3, *p* < 0.05); Appendix C Figure A2c). Constipation appears to be the most affected symptom, with an increase from baseline in most consolidation cycles of around 10 points (baseline: 0 (range: −1.97–1.97, *p* > 0.05); cycle 6: 6.808 (range: 2.77–10.84, *p* < 0.05); Appendix C Figure A2f).

### 3.4. Quality of Life during the Maintenance Period

#### 3.4.1. GHS/QoL

**Figure 1:**
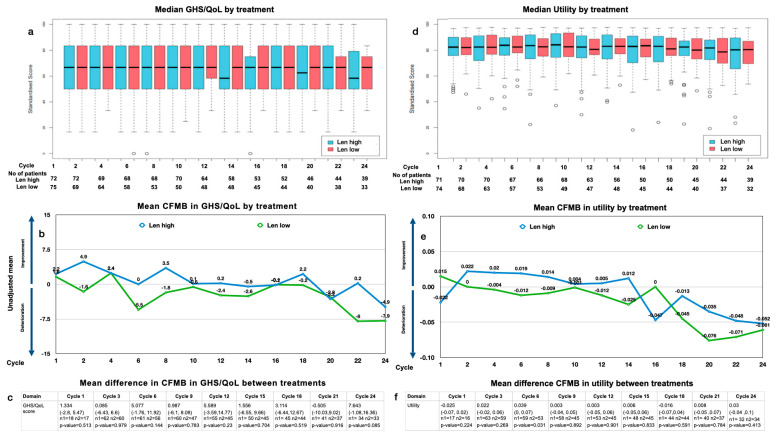
GHS/QoL and utility: (**a**) median GHS/QoL by treatment and cycle and patient number analyzed by treatment and cycle; (**b**) mean change from maintenance baseline (CFMB) in GHS/QoL by treatment and cycle; (**c**) mean CFMB between treatments (mean (range), n = patients analyzed, *p*-value); (**d**) median utility by treatment and cycle and patient number analyzed by treatment and cycle; (**e**) mean CFMB in utility split by treatment and cycle; (**f**) mean difference in CFMB between treatments (mean (range), n = patients analyzed, *p*-value).

The overall median GHS/QoL scores were generally constant over 2 years of maintenance (Figure 1a). The mean change from maintenance baseline (CFMB) in the GHS/QoL over time within the arms was mostly <5 but showed a tendency for deterioration during the last cycles in both arms (Figure 1b).

Analyzing the median GHS/QoL by treatment arm, we found no significant difference over time (median: 68 for Len high (range: 16–100) and 68 for Len low (range: 34–100) at baseline; 58 for Len high (range: 16–100) and 68 for Len low (range: 34–83) at 2 years; both *p* > 0.05). Analyzing the mean difference in the CFMB between treatment arms, we found no significant difference between Len high and Len low over 2 years of treatment (*p* > 0.05) (Figure 1c). The MID of 5 is surpassed slightly at cycles 6 and 12, yet it drops quickly to surpass 5 again at cycle 24.

#### 3.4.2. Utility Values

The overall median utility values were constant at most time points, with a slight downward trend toward the end of year 2 with a mean change of 0.04 (Figure 1d). The mean CFMB over time within the arms showed a slight decline in the utility over 2 years of maintenance (Figure 1e).

Analyzing the median utility by treatment arm over time, we observed the utility for patients with Len high to be generally similar to that of those with Len low (median: 0.7 for Len high (range: 0.35–0.95) and 0.71 for Len low (range: 0.36–0.96) at baseline; 0.68 for Len high (range: 0.22–0.95) and 0.68 for Len low (range: 0.27–0.96) after 2 years; *p* > 0.05). Analyzing the mean difference in the CFMB between the treatment arms, we found no significant difference between Len high and Len low over 2 years of treatment (*p* > 0.05) (Figure 1f).

#### 3.4.3. Function and Symptom Scales, Single Items

Analyzing the medians and CFMB over time confirmed that there were no obvious differences for many of the functional scale domains, such as role functioning, cognitive functioning, or emotional functioning. Similarly, symptom scales, like fatigue or nausea and vomiting, were constant and low for all cycles. The single-item scores for insomnia, appetite loss, constipation, and dyspnea were consistent.

**Figure 2:**
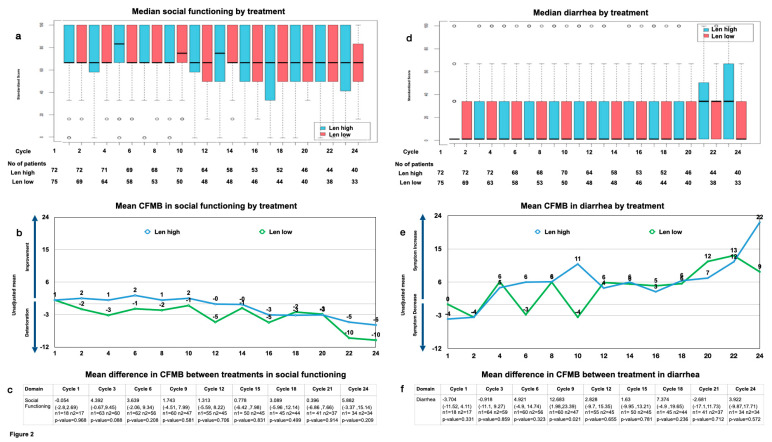
Social functioning (**a**) median by treatment and cycle and patient number analyzed by treatment and cycle; (**b**) mean change from maintenance baseline (CFMB) by treatment and cycle; (**c**) mean difference between treatments in CFMB (mean (range), n = patients analyzed, *p*-value) diarrhea; (**d**) median by treatment and cycle and patient number analyzed by treatment and cycle; (**e**) mean CFMB by treatment and cycle; (**f**) mean difference between treatments in CFMB (mean (range), n = patients analyzed, *p*-value).

The most notable overall changes included social functioning, which showed a significant downward trend over time in both arms, leading to a mean CFMB of approximately 10 points at the end of year 2, thus surpassing the MID (baseline: 0.952 (range: −0.4–2.3); end of year 2: −10.119 (range: −16.53–−3.71); *p* < 0.05) (Figure 2a,b). The mean difference in the CFMB between the treatment arms showed no significant difference until the end of year 2, when the difference surpassed the MID (baseline: −0.054 (range: −2.7–2.69, *p* > 0.05); end of year 2: 5.882 (range: −3.37–15.14, *p* > 0.05)) (Figure 2c).

Also, diarrhea showed a consistent increase, leading to a significant mean CFMB at the end of year 2 exceeding the MID within the treatment arms (baseline: −1.905 (range: −5.78–1.97); end of year 2: 16.071 (range: 5.72–26.42), *p* < 0.05)) (Figure 2d,e). The mean difference in the CFMB between the treatment arms showed the MID to surpass 5 at cycles 9 and 18, yet it drops quickly to surpass the MID again at cycle 24. Notably, diarrhea increased slightly more for Len high by the end of year 2 without reaching statistical significance (mean difference in the CFMB between treatments for diarrhea at baseline: −3.704 (range: −11.52 −4.11, *p* > 0.05), and that at cycle 24: 3.922 (range: −9.87–17.571, *p* > 0.05) (Figure 2f).

### 3.5. Subgroup Analysis

To analyze how external factors such as disease control, adverse events (AEs), or the burden of an extended treatment time could affect HR-QoL, we looked at utility in relation to the remission status, severity of adverse events, or time on treatment. The subgroup analysis revealed that neither the disease response ((s)CR vs. less than CR), duration of treatment, nor adverse events (AEs grade 1/2, 3/4 vs. none) affected the HR-QoL or utility (see Appendix D for further details).

### 3.6. Missing Data

The number of patients on trial was similar in both arms (Figure 2). This indicates that the extent of the missing data was comparable and not missing at random, which eliminates bias in the HR-QoL toward either treatment arm (see Appendix A for further details on reasons for dropping out).

## 4. Discussion

Despite advances in the overall survival, multiple myeloma remains an incurable disease that substantially affects patients’ HR-QoL. To date, the LenaMain trial is the only clinical trial to show that a higher dosage of lenalidomide maintenance post-transplant benefits patients by extending the time of remission. Here, we show that a higher Len dose does not substantially impair HR-QoL, and patients can therefore maintain the high level of functioning exhibited at study entry even at high dosages. The EORTC QLQ-C30 scores were high for functioning and low for symptoms and were similar to those of the general population, which shows a GHS/QoL between 76 and 79 [25]. Thus, our study population was largely asymptomatic and had a high level of functioning at study entry.

Consolidation after autologous stem cell transplantation has long been a point of discussion [26,27]. In our study, consolidation with six cycles of 25 mg of lenalidomide was feasible and no unexpected toxicity occurred. Other studies have shown significant survival benefits from lenalidomide, as well as bortezomib-containing consolidation therapy, while maintaining the HR-QoL (14 and 13 months, respectively, and both with GHS/QoL values around 70) [28,29]. Thus, our HR-QoL data confirm these findings by showing very little variation in the HR-QoL, utility, and functional and symptom scores. Some domains, however, show some variation during the first cycles of consolidation, most likely reflecting some uncertainty about the upcoming therapy amid a clinical trial.

During the lenalidomide maintenance, both the GHS/QoL and utility scores were relatively stable. The overall scores declined slightly but only exceeded the previously assigned MID at the end of year 2. This suggests that the high levels of the HR-QoL and utility observed at study entry were maintained, which is consistent with previous results showing no deterioration in the HR-QoL despite continued lenalidomide maintenance [19].

When we compared the GHS/QoL and utility between both arms, we found no significant difference between Len high and Len low. Also, we found consistent values for the function and symptom scales, as well as for the majority of single items, over the course of the trial, without substantial differences between the treatment arms. Noticeable exceptions were an increase in diarrhea and a decrease in social functioning. Both exceeded the pre-specified MID in both arms by the end of year 2.

Diarrhea is a common side effect with Len treatment that can severely impact HR-QoL and may lead to the unnecessary discontinuation of therapy [30]. Here, we show that diarrhea as a symptom exceeded the MID but was independent of the Len dosage. Hence, fear of diarrhea associated with decreased HR-QoL should not prevent physicians from prescribing high-dose Len MT. Instead, more supportive care should be applied. Bile acid malabsorption has recently been shown to cause Len-associated diarrhea, and treatment with a bile acid sequestrant is recommended in these cases [31]. Unfortunately, these data were not yet available during our study, but they may enable more patients in the future to continue long-term treatment with Len high, achieve better disease control, and improve their HR-QoL.

The other noticeable exception we observed was an overall downward trend in social functioning without any difference between the treatment groups until the end of the 2-year observational period, when the MID for both the CFMB and CFMB between the groups was surpassed. Despite disease stability and the absence of acute treatment-related toxicity, reduced social functioning is often related to a persistent and cumulative symptom burden [32]. Jordan et al., reported that social functioning was strongly affected by even moderate symptom levels [33]. And while patients may achieve long-term disease control with lenalidomide maintenance, the median time to relapse is 26.9 months [34]. Therefore, symptoms may begin to accumulate again after 2 years before meeting the criteria of progressive disease, further exacerbating social function deficits over time. Moreover, patients’ concerns about controlling their bowels could certainly impair their social functioning, connecting both items.

Adverse effects, like treatment-related toxicities (e.g., rash, fatigue, diarrhea/constipation, neuropathy) or infections, can significantly affect HR-QoL. Alleviation of these toxicities could be reflected by improvements in the HR-QoL, just as the aggravation of these toxicities might impair the HR-QoL. Hematologic toxicities, especially grade ≥ 3 neutropenia and infections, were a common side effect in the LenaMain trial and were observed in 34.6%, 24.3%, and 12.8% of the Len-high arm during the first, second, and third years, respectively, whereas the incidence in the Len-low arm remained constant at about 9%. We found that the GHS/QoL and utility in both treatment groups were not affected by high-grade toxicity. Similarly, AEs did not lead to more patients discontinuing lenalidomide maintenance. This result is consistent with reports from other clinical trials showing that regimens that prolong progression-free survival also provide sustained stable and improved HR-QoL, suggesting that a clinically meaningful improvement in relevant symptoms outweighs the adverse effects of treatment [35,36].

## 5. Relevance

Continuous therapy for multiple myeloma is often associated with a plethora of side effects. Thus, physicians might hesitate to use lenalidomide at the recommended dose, especially out of fear of infections or neuropathy. Some myeloma patients might also prioritize quality of life over a long-term response, further prompting dose reductions. We were the first to show that high-dose lenalidomide extends the progression-free survival but is associated with an increase in grade 3/4 infections in comparison to lower dosages of lenalidomide. Here, we show that a higher Len dosage is well tolerated, and the quality of life is not dose-dependent. Severe AEs, like infections, also did not impact the long-term quality of life. This result argues for titrating Len maintenance up to the best-tolerated dose in order to reach the optimal efficacy. Others have also argued for an individualized approach based on the disease characteristics, response to induction and ASCT (or even non-ASCT consolidation approaches, such as CAR-T-cell therapy or bispecific antibodies), as well as patient preferences [37]. Newer data from the DETERMINATION trial have also taken QoL into account [38]. This trial investigated the effect of adding ASCT to triplet therapy (lenalidomide, bortezomib, and dexamethasone) followed by lenalidomide maintenance and showed superior progression-free survival in the transplant group. Although the investigators observed a transient but clinically meaningful decrease in the quality of life associated with ASCT, the mean QoL scores subsequently recovered. In fact, the mean improvements from the maintenance baseline remained numerically higher in the post-transplant group throughout the maintenance therapy. This further supports that a more effective therapy results in better QoL, which emphasizes that personalized approaches are paramount when considering the toxicities and treatment effects on the QoL. But our results could also apply to the relapsed setting. With the advent of triplet combinations for relapsed patients, physicians might use dose-reduced Len to prevent side effects. By establishing high-dose Len maintenance as independent of grade 3/4 adverse events, our data also argue for a higher dose in the relapsed setting in order to achieve fast and durable responses.

## 6. Conclusions

The lenalidomide dosage during long-term maintenance had no impact on the quality of life in multiple myeloma patients. Instead, high baseline scores for global health and utility were maintained throughout the trial, without differences between the treatment arms. Thus, the HR-QoL was not governed by the higher rate of infectious toxicity caused by high-dose lenalidomide, which supports the feasibility of continuous Len treatment with a dose titrated on an individual basis and tailored to patients’ HR-QoL.

## Figures and Tables

**Table 1:** Patient characteristics (GHS: global health status; QoL: Quality of life; n: number; SD: standard deviation).

Characteristic	Level	Overall (n = 188)			Lenalidomide 25 mg n = 94		Lenalidomide 5 mg n = 94
Age median, range		59.00 (34.00–73.00)			59.00 (34.00–72.00)		59.00 (34.00–73.00)
Gender n (%)	Male	116 (61.70)			56 (59.57)		60 (63.83)
β-2-Microglobulin levels n (%)	≥2.5	54 (28.72)			28 (29.79)		26 (27.66)
	<2.5	134 (71.28)			66 (70.21)		68 (72.34)
Prior therapies n (%)	1	156 (82.98)			76 (80.85)		80 (85.11)
	2	32 (17.02)			18 (19.15)		14 (14.89)
Radiotherapy n (%)	1	65 (34.57)			30 (31.91)		35 (37.23)
	2	123 (65.43)			64 (68.09)		59 (62.77)
EORTC QLQ-C30 domain or utility		Baseline			Maintenance Baseline		
		Overall	Lenalidomide 25 mg	Lenalidomide 5 mg	Overall	Lenalidomide 25 mg	Lenalidomide 5 mg
Utility—mean (SD)	Utility	0.70 (0.20)	0.69 (0.21)	0.71 (0.20)	0.71 (0.18)	0.69 (0.20)	0.72 (0.15)
GHQ/Qol score—mean (SD)	GHS/QoL	65.78 (19.90)	64.81 (19.34)	66.76 (20.51)	65.25 (19.66)	63.08 (21.30)	67.33 (17.85)
Functional domain scores—mean (SD)	Cognitive functioning	84.72 (20.31)	84.81 (22.71)	84.63 (17.71)	78.23 (23.38)	79.63 (22.42)	76.89 (24.34)
	Emotional functioning	75.15 (23.77)	76.73 (23.21)	73.58 (24.35)	72.05 (22.39)	71.99 (23.71)	72.11(21.20)
	Physical functioning	77.01 (20.38)	76.00 (20.96)	78.02 (19.84)	77.14 (18.22)	76.94 (19.54)	77.33 (16.98)
	Role functioning	66.11 (30.41)	65.00 (29.78)	67.22 (31.15)	64.17 (26.27)	64.12 (26.78)	64.22 (25.95)
	Social functioning	69.17 (27.94)	69.44 (29.38)	68.89 (26.58)	71.88 (26.66)	70.83 (29.57)	72.89 (23.69)
Symptom domain scores—mean (SD)	Fatigue	31.64 (24.03)	32.35(23.77)	30.93 (24.40)	32.99 (23.57)	34.26 (23.86)	31.78 (23.38)
	Nausea and Vomiting	3.61 (12.37)	2.41 (9.20)	4.81 (14.83)	4.76 (11.21)	3.70 (9.37)	5.78 (12.70)
	Pain	29.17 (27.85)	33.15 (28.65)	25.19 (26.59)	29.02 (24.21)	31.02 (25.53)	27.11 (22.89)
Single item scores—mean (SD)	Appetite Loss	7.96 (19.42)	7.04 (19.02)	8.89 (19.86)	8.84 (18.43)	10.65 (20.80)	7.11 (15.78)
	Constipation	7.22 (19.39)	7.41 (20.47)	7.04 (18.36)	14.29 (26.46)	17.59 (30.11)	11.11(22.15)
	Diarrhoea	8.70 (19.09)	7.04 (16.94)	10.37 (20.99)	12.70 (22.86)	9.72 (21.26)	15.56 (24.10)
	Dyspnoea	20.86 (26.19)	20.37 (26.77)	21.35 (25.75)	22.90 (24.61)	20.37 (22.76)	25.33 (26.19)
	Financial Difficulties	23.22 (30.23)	20.97 (28.60)	25.47 (31.79)	22.15 (30.14)	18.06 (26.79)	26.13 (32.77)
	Insomnia	23.15 (29.08)	23.33 (31.39)	22.96 (26.74)	28.08 (28.95)	28.64 (30.50)	27.56 (27.60)

**Figure A3:**
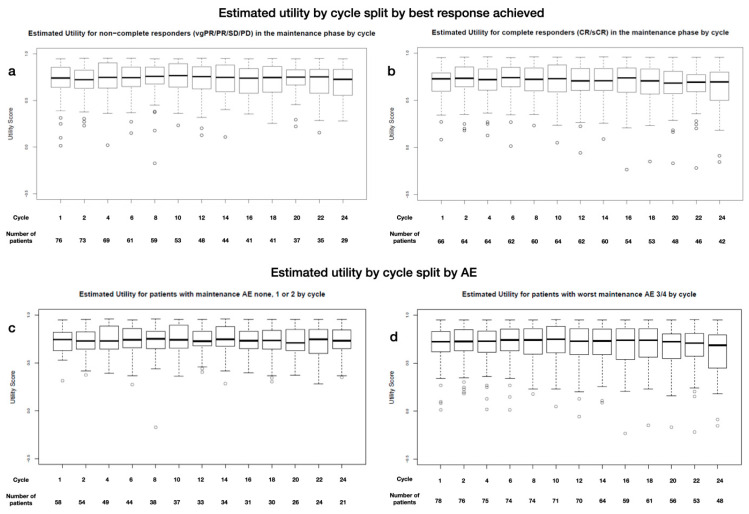

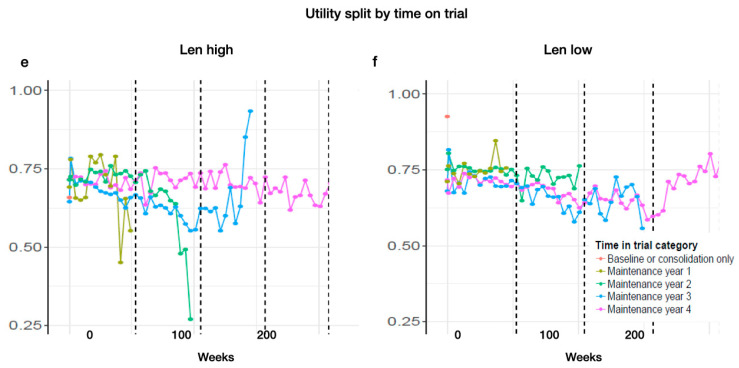
Estimated utility during MT by cycle and grouped by (**a**) non-complete responders (vgPR, PR, SD) and (**b**) complete responders (CR, sCR), by severity of experienced AEs: (**c**) grade 1 and 2 or (**d**) 3 and 4, and by time of treatment for (**e**) Len high and (**f**) Len low (color coding for time and MT treatment).

## Data Availability

Data analyses were performed by KKS Düsseldorf, Germany, and Bresmed, Sheffield, the UK. All authors had access to the primary clinical trial data.

## Appendix A. Materials and Methods

### Appendix A.1. Study Design and Treatment

The LenaMain trial was an investigator-initiated, randomized, open-label, phase-III trial conducted in six hospitals in Germany, which recruited patients from 4 June 2009 to 1 February 2015 (ClinicalTrials.gov NCT00891384). Details were previously published [1]. Briefly, 194 patients were enrolled, and 188 patients were randomized 1:1 to either receive lenalidomide at a 5 mg (Len-low) or 25 mg (Len-high) maintenance treatment (Len MT) after first-line therapy with autologous stem cell transplant. Patients in both treatment arms first entered a uniform consolidation period and received six cycles of lenalidomide at 25 mg (for 21 days every 28 days), after which they started their respective maintenance therapies.

### Appendix A.2. HR-QoL Assessments and Endpoint

The EORTC Quality of Life Questionnaire, EORTC QLQ-C30 (QLQ-C30), is the most frequently used instrument for cancer-specific HR-QoL assessment (scale range: 0–100) in myeloma patients [2,3,4]. It includes 30 items comprising one global health status (GHS) scale, five functional scales (physical, role, emotional, cognitive, and social functioning), three symptom scales (fatigue, nausea and vomiting, and pain), and six single items (dyspnea, insomnia, appetite loss, constipation, diarrhea, and financial difficulties). Higher scores represent better GHS and functioning but also greater (i.e., worse) symptoms.

The primary patient-reported outcome (PRO) hypothesis presumed similar HR-QoL for the two different lenalidomide doses during maintenance. Patients completed the questionnaires (paper) at baseline (day 1 of cycle 1) and on day 1 of each cycle. The questionnaire was scored using guidelines recommended by the EORTC [5].

Institutional review boards of all participating institutions approved the study protocol. The EORTC approved the use of the QLQ-C30 questionnaire. All patients provided written informed consent.

### Appendix A.3. Assessing Raw Data

First, a table was created that summarized the number of missing observations in the data at each cycle based on the randomized number in each group. A comparison to the expected number of patients in each cycle was produced taking into consideration the number of patients still ongoing in the trial if the necessary data on the progression dates were available (given that the QoL was not recorded following the PD confirmation visit).

The approximate expected number of patients at each cycle was based on the patient’s event-free survival (EFS) date, where the time at which the EORTC QLQ-C30 questionnaires were expected to be collected was between the first consolidation cycle data and the EFS date. The expected number of questionnaires was calculated based on the assumption that each patient completed a questionnaire every 4 weeks until their EFS date. If one or more of these dates was missing, then this was dealt with on a case-by-case basis, taking what was considered the most reasonable approach for defining the period in which the patient should contribute questionnaires.

For the maintenance baseline, the initial suggestion was to use maintenance cycle 1 (MC1) as the baseline value, with consolidation visit records as a backup. After investigating the data, to make the most use of the records at MC1, the consolidation visit was taken as the maintenance baseline if available, with the MC1 values being used if no consolidation record was available (performed on a case-by-case basis for each domain). This led to totals of 72 and 75 records on treatment arms 1 (25 mg) and 2 (5 mg), respectively (out of approximate expected numbers of 91 and 88).

### Appendix A.4. Calculating QLQ-C30 Scores

The EORTC QLQ-C30 assesses QoL based on five functional scales, three symptom scales, six single items, and a global health status/QoL scale, defined based on specific combinations of the 30 individual questions of the QLQ-C30 shown in Table A1, along with the corresponding range (r) of each scale.

**Table A1:** EORTC QLQ-C30 scoring.

Category	Scale	Code	Range (*r*)	EORTC QLQ-C30–Corresponding Questions
Global health status/QoL	Global health status/QoL	QL	6	29, 30
Functional scales	Physical functioning	PF	3	1–5
	Role functioning	RF	3	6, 7
	Emotional functioning	EF	3	21–24
	Cognitive functioning	CF	3	20, 25
	Social functioning	SF	3	26, 27
Symptom scales	Fatigue	FA	3	10, 12, 18
	Nausea and vomiting	NV	3	14, 15
	Pain	PA	3	9, 19
Single items	Dyspnea	DY	3	8
	Insomnia	SL	3	11
	Appetite loss	AP	3	13
	Constipation	CO	3	16
	Diarrhea	DI	3	17
	Financial difficulties	FI	3	28

Legends: patient characteristics (GHS: global health status; QoL: quality of life; CFB: change from baseline; N/n: number; SD: standard deviation; BL: baseline). Note: the range (r) is the difference between the maximum and minimum possible values of the raw score (e.g., for an item scored 1–4, r = 3); all items except for those contributing to the global health status/QoL scales are scored from 1 to 4.

The QLQ-C30 scoring manual describes the process for calculating the scores from the individual questionnaire responses:

- Raw scores (RSs) are calculated as the mean of the responses that belong to each scale or item (e.g., for the physical function, this would be the mean of the responses for questions 1–5);
- Standardized scores (Ss) are calculated using a transformation such that they vary at an interval from 0 to 100.

1. For functional scales: $S=100\times \left\{1-\frac{\left(RS-1\right)}{r}\right\}$;
2. For symptom scales, single items, global health status/QoL scales: $S=100\times \left\{\frac{\left(RS-1\right)}{r}\right\}$.

Note: If items are missing, then the following process should be followed:

3. If at least half of the items that contribute to a scale have been answered, then calculate the score as above, ignoring the missing items;
4. If more than half are missing, then the scale is set to missing;
5. Single items will always be set to missing.

### Appendix A.5. Utility Mapping

Utility values were calculated based on the QLQ-C30 questionnaire responses. The mapping described by Longworth et al., was used to derive utilities based on a UK tariff [6]. This mapping algorithm was chosen based on the range of statistical methods considered in this publication for mapping, the range of EQ-5D values available in the population used for the mapping, and the consistency of the questionnaire version.

### Appendix A.6. Analyses Performed

The methods were motivated by the key aims of this work and a recent publication that assesses a similar topic [7]. These were conducted using a combination of software, including R v3.3.3, SAS 9.4, and Microsoft Excel Version Professional Plus 2016. Statistical analysis was performed by Bresmed, Sheffield, the UK.

### Appendix A.7. Change from Baseline

The mean change from baseline was assessed for both the consolidation and maintenance periods, plotted against time in weeks. As the cycles were 4 weeks long, this resulted in QLQ-C30 records every 4 weeks, as the questionnaires were collected at the start of each cycle. The corresponding weeks are the number of weeks at the start of each cycle; for instance, consolidation cycle 1 has a QLQ-C30 record at the start of the cycle that corresponds to week 1, and the second cycle therefore corresponds to week 5.

### Appendix A.8. Minimally Important Difference (MID)

Despite the appeal of the notion, there is no universal MID. At both the group and individual level, the MID may depend on the clinical context and decision at hand, the baseline from which the patient starts, and whether they are improving or deteriorating [8]. Moreover, as a statistically significant change in the HR-QoL is not necessarily important to the patient, the MID is a more useful way of interpreting changes in the HR-QoL scores for clinical decision making [9].

We defined the MID threshold for clinically meaningful change from baseline a priori for each PRO based on the published literature at ≥5 points. Kvam et al., in a longitudinal study, previously concluded that a difference of 6–17 points (0–100 scale) in the EORTC QLQ-C30 represents a clinically meaningful change in patients with MM [10]. Kvam et al., also suggested 5–6 to be a small change, and 11–15 to be a medium change [11]. For the change from baseline within groups, Cocks et al., with longitudinal contrasts, previously determined a mean increase for the global quality of life at 6.4 and a mean decrease at −9.5, a mean increase for diarrhea at 6.2 and mean decrease at −4.1, and for social functioning, a mean increase at 6.2 and mean decrease at −8.7 to be significant [12]. For mean differences between groups, Cocks et al., with cross-sectional contrasts, determined a mean difference of 7.22 for the global quality of life, 4.4 for diarrhea, and 7.82 for social functioning [13]. Thus, an MID of ≥5 was a deliberate choice and based on the fact that patients eligible for maintenance are often asymptomatic and seek to maintain their HR-QoL in addition to extending disease control [14,15].

### Appendix A.9. Subgroup Analysis

To assess the impact of the responses on utility during maintenance, two subgroups were selected: complete responders (patients who achieved a stringent complete response (sCR) or complete response (CR)) and non-complete responders (very good partial response (vgPR), partial response (PR), stable or progressive disease).

To assess the impact of adverse events on the utility and GHS/QoL during maintenance, patients were grouped into those with adverse events (AEs) of grade 1/2 according to the common terminology criteria (CTC) of adverse events, those with AEs of grade 3/4, or those without AEs.

Further, we wondered whether the patients’ time on treatment led to differences in the HR-QoL; thus, an exploration was conducted for the utility and the GHS/QoL scale. The patients’ time on treatment was based on the latest HR-QoL cycle record.

### Appendix A.10. Study Discontinuations

**Table A2:** Reasons for Discontinuation.

	25 mg	5 mg
**Discontinuation**	61 (65%)	74 (78%)
Due to progressive disease	27 (29%)	42 (45%)
Due to AE	27 (29%)	26 (28%)
Due to death	3 (3%)	1 (1%)
Due to refusal	4 (4%)	5 (5%)
Median time (range) until EOT (months)	26.8 (0.5–87)	22.9 (0.3–69)

### Appendix A.11. Dose Reductions

**Table A3:** Reasons for Dose Reduction.

Dose Reduction Due to	N = 337
Neutropenia	187 (56)
Thrombocytopenia	31 (9)
Constitutional symptoms	24 (7)
Infection	17 (5)
Dermatological AEs	14 (4)
New primary malignancy	13 (4)
Neurological AEs	13 (4)
Patient request	9 (3)
Anemia	9 (3)
Renal AEs	6 (2)
Gastrointestinal + Liver AEs	5 (2)
Death	4 (1)
Pulmonal AEs	3 (1)
Cardiac AEs	2 (1)
Bleeding	0 (0)

Abbreviations: AE: adverse event, EOT: end of treatment.

## Appendix D. Subgroup Analysis

### Results

To analyze how external factors, such as disease control, adverse events (AEs), or the burden of an extended treatment time, could affect HR-QoL, we looked at utility in relation to the remission status, severity of adverse events, or time on treatment.

The boxplots show very little difference between the utility for the complete responder and non-complete responder subgroups (median: 0.65 at baseline, 0.6 at year 1, and 0.6 at year 2, and 0.65 at baseline, 0.66 at year 1, and 0.6 at year 2, respectively, *p* > 0.05) (Appendix D Figure A3a,b). Similarly, experience of light (maximum grade: 1 + 2) or severe (maximum grade: 3 + 4) AEs did not affect utility. The boxplots show no clear differences between cohorts (0.65 at baseline, 0.6 at year 1, and 0.6 at year 2, and 0.65 at baseline, 0.66 at year 1, and 0.6 at year 2, respectively, *p* > 0.05) (Appendix D Figure A3c,d).

Looking at the time on treatment, categorized by 1, 2, 3, and 4 years, we find a tendency for those patients who were on treatment the longest to show reasonably consistent and slightly higher utility values across the whole time (Len-high MT year 1: baseline: 0.69; year 1: 0.56. MT year 2: baseline: 0.71; year 1: 0.71; year 2: 0.26. MT year 3: baseline: 0.64; year 1: 0.66; year 2: 0.63; year 3: 0.93. MT year 4: baseline: 0.70; year 1: 0.71; year 2: 0.74; year 3: 0.73; year 4: 0.71. Len-low MT year 1: baseline: 0.69; year 1: 0.56. MT year 2: baseline: 0.71; year 1: 0.70; year 2: 0.26. MT year 3: baseline: 0.63; year 1: 0.65; year 2: 0.63; year 3: 0.93. MT year 4: baseline: 0.71; year 1: 0.65; year 2: 0.74; year 3: 0.73; year 4: 0.70) (Appendix D Figure A3e,f).
